# A Tale of Two Meckel’s: small bowel obstruction secondary to Meckel’s Diverticulum

**DOI:** 10.1093/jscr/rjab037

**Published:** 2021-03-15

**Authors:** Nargus Ebrahimi, Rakesh Quinn, Yi Liang, Richard Curran

**Affiliations:** General Surgery, Blacktown Hospital, Blacktown, NSW, Australia; General Surgery, Blacktown Hospital, Blacktown, NSW, Australia; General Surgery, Blacktown Hospital, Blacktown, NSW, Australia; General Surgery, Blacktown Hospital, Blacktown, NSW, Australia

## Abstract

We present two rare cases of small bowel obstruction (SBO) secondary to Meckel’s diverticulum (MD) where the mechanism of obstruction was not readily apparent. Both were cases of virgin abdomen with pre-operative CT scans demonstrating SBO without a clear underlying cause or mass. Diagnostic laparoscopy was performed, which established the underlying cause to be MD, and laparoscopic-assisted resection was undertaken to resect small bowel and perform a side-to-side stapled anastomosis. We subsequently describe the different mechanisms by which MD can cause obstruction as described in the literature.

## INTRODUCTION

Meckel’s diverticulum (MD) is uncommon as a finding, occurring in 2% of the population, while complicated or symptomatic cases of MD are even more rarely encountered. The rule of two’s succinctly summarizes the key features of this embryological variant: ~2% prevalence, 2 inches in length, 2 feet proximal to the ileocaecal valve located on the antemesenteric border of the ileum, some (15–50%) containing 2 types of heterotopic mucosa (gastric or pancreatic) and most cases presenting before the age of 2 [1, 2]. We describe two cases of MD causing small bowel obstruction (SBO) in two adults with a virgin abdomen where the mechanism of obstruction was not readily apparent. Additionally, of note, these two patients presented over 2 consecutive days at the same institution, a coincidental adherence to the oft-quoted ‘rule of twos’.

**Figure 1:**
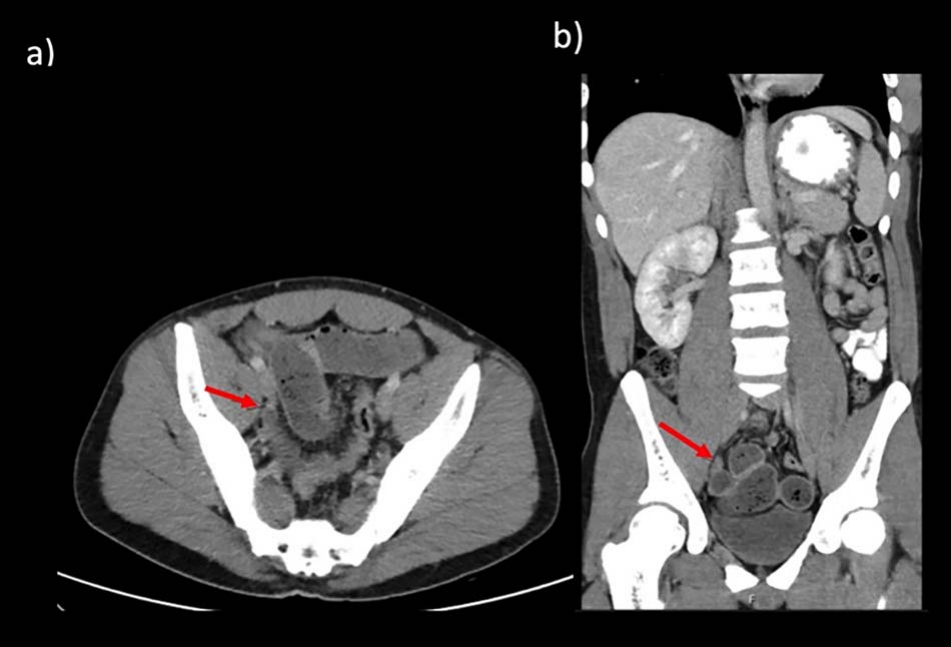
CT abdomen findings from Cases 1 and 2 showing distal SBO with the transition point in the pelvis. (**a**) Case 1 CT axial view. (**b**) Case 1 CT coronal view.

## CASE REPORT

**Figure 2:**
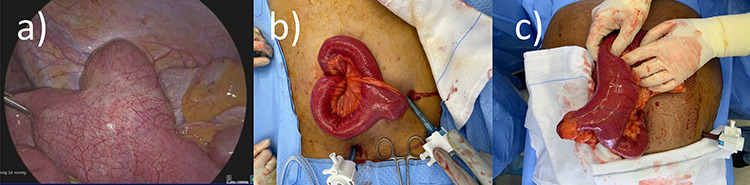
Operative findings from Cases 1 and 2 of MD at the transition point. (**a**) Case 1 laparoscopic image showing MD. (**b**) Case 1 operative image of MD (after conversion to mini-laparotomy). (**c**) Case 2 operative image of MD (after conversion to mini-laparotomy).

Case 1 is a 24-year-old male with a virgin abdomen who presented with a 3-day history of crampy abdominal pain and vomiting. He was haemodynamically normal and afebrile. He had tenderness in the lower abdomen and no peritonism. There was a moderate leukocytosis with a WCC of 12.7 × 10^9^/L. An abdominal CT demonstrated distal SBO and a transition point located in the right pelvis and a small amount of free fluid (Fig. 1). The appendix was identified as normal. On Day 0 of his admission, he underwent a diagnostic laparoscopy. Intra-operatively, an MD was found at the site of the transition point between small bowel dilated proximally and collapsed distally (Fig. 2). Enteric contents were thickened raising the possibility of a faecolith. Macroscopically, the MD appeared to be normal, with no features of intussusception, volvulus or inflammation at the site. The MD was exteriorized through a mini-laparotomy and small bowel resection with a side-to-side stapled anastomosis was performed. Histopathology revealed MD with no features of inflammation or ectopic mucosa.

**Figure 3:**
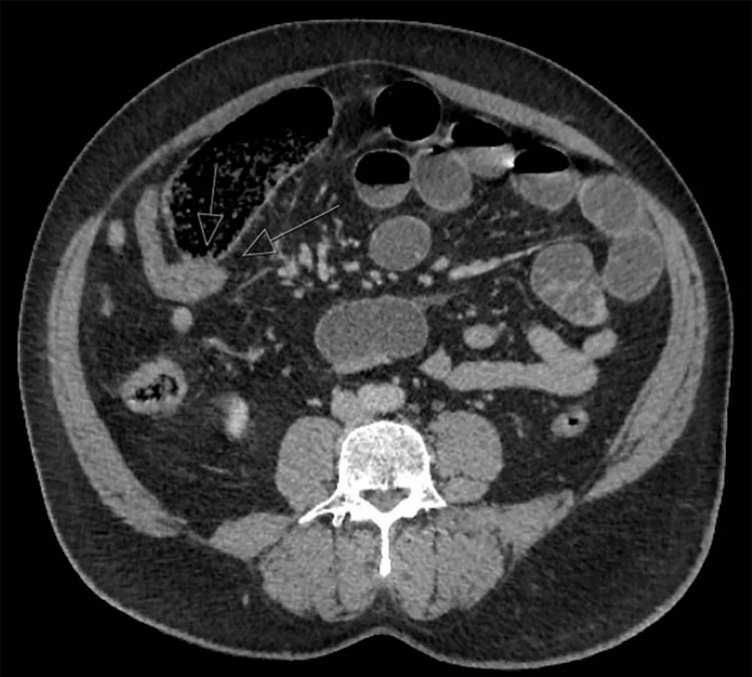
Case 2 CT abdomen axial view showing a distal SBO.

Case 2 is a 56-year-old male, with a virgin abdomen, who had 2 days of crampy abdominal pain, vomiting and obstipation. He was haemodynamically normal and afebrile. He had a distended abdomen with generalized tenderness. Blood tests showed an elevated lactate of 3 and WCC of 11.7 × 10^9^. CT demonstrated distal SBO, with distension of small bowel up to 6 cm (Fig. 3). He underwent a diagnostic laparoscopy with an identification of MD at the transition point, subsequently exteriorized through a mini-laparotomy (Fig. 2). The apex of the MD was tethered to the mesentery through a band containing the diverticular blood supply. Small bowel resection and anastomosis was performed. Histopathology showed MD with acute inflammation, haemorrhage and necrosis, and no ectopic tissue.

## DISCUSSION

MD is an example of a true diverticulum, encompassing all layers of the small bowel wall, with a blood supply derived from a terminal branch of the superior mesenteric artery [3]. While initially reported by German surgeon Wilhelm Fabricus Hildenus in 1598, MD is eponymous for German anatomist Johann Friedrich Meckel the Younger, who first described its embryogenesis in 1809 [1]. MD arises due to the failure of closure of the omphalomesenteric duct during the 5th to 7th weeks of development [4].

Complicated MD occurs in 4% of cases and associated features include age less than 50, male sex, abnormal mucosa and length >2 cm [5]. Complications include obstruction, inflammation, perforation, bleeding or malignancy [1, 5]. Multiple mechanisms may precipitate obstruction, with the most common events being volvulus or intussusception [1, 5]. The presence of embryonic bands attaching the MD to the umbilicus or to the mesentery is commonly implicated. An omphalomesenteric band, which connects the MD to the umbilicus, can lead to volvulus or entrapment of bowel. A mesodiverticular band is one that is attached to the diverticulum and ileal mesentery and may directly compress the ileum or create an opening for internal herniation.

Intussusception can also occur, particularly when the diverticulum is short and thickened with inflammation, ectopic tissue or tumour, acting as a lead point [3]. Other mechanisms include previous diverticulitis episodes causing band adhesions, acid secretion by ectopic mucosa leading to luminal stenosis, occlusion by faecoliths, enteroliths or bezoars, or incarceration within an inguinal hernia called Littre’s hernia [1, 6].

The diagnosis of MD as the cause of SBO is often not made until the operation. CT is very accurate in identifying an obstruction, although it has poor sensitivity and specificity in detecting MD. For instance, pre-operative diagnosis with CT was made in only 50% in a recent case series of MD causing SBO. Radiological features suggesting this diagnosis include dilated small bowel loops with a transition point at or near midline, presence of a blind-ending tubular pouching of the distal ileum located at the terminal branch of the SMA with or without inflammation or enterolith. Relevant negative findings include a normal appendix and no previous surgery [7].

In both cases, diagnostic laparoscopy was used to establish and treat the cause of SBO. Unlike cases described in the literature where a specific mechanism is associated with MD causing obstruction, in the two cases we present, the exact mechanism was not directly observed. In case 1, the enteric contents were quite thickened; the possibility of an enterolith could not be excluded, although this was not demonstrated on CT scan. The microscopic diagnosis was also not remarkable, and therefore, the sequence of events leading to obstruction with the transition point is unclear. In the second case, it appeared that a band or adhesions caused tethering of the apex of the MD to the ileal mesentery, which served as the site of the transition point. There was inflammation noted on histopathology; therefore, it is likely that this contributed to the obstruction, although this could be a secondary phenomenon if the adhesions caused volvulus leading to obstruction, as described in another case [8].

## CONFLICT OF INTEREST STATEMENT

The authors declare no conflict of interest.

## FUNDING

None.

## References

[1] Lindeman RJ, Soreide K. The many faces of Meckel's diverticulum: update on management in incidental and symptomatic patients. Curr Gastroenterol Rep 2020;22:3. 31930430 10.1007/s11894-019-0742-1

[2] Rivas H, Cacchione RN, Allen JW. Laparoscopic management of Meckel's diverticulum in adults. Surg Endosc 2003;17:620–2. 12582775 10.1007/s00464-002-8613-4

[3] Williams RS. Management of Meckel's diverticulum. Br J Surg 1981;68:477–80. 7018640 10.1002/bjs.1800680712

[4] Yoo JH, Cerqueira DS, Rodrigues AJ Jr, Nakagawa RM, Rodrigues CJ. Unusual case of small bowel obstruction: persistence of vitelline artery remnant. Clin Anat 2003;16:173–5. 12589674 10.1002/ca.10086

[5] Park JJ, Wolff BG, Tollefson MK, Walsh EE, Larson DR. Meckel diverticulum: the Mayo clinic experience with 1476 patients (1950-2002). Ann Surg 2005;241:529–33. 15729078 10.1097/01.sla.0000154270.14308.5f PMC1356994

[6] Clark JK, Paz DA, Ghahremani GG. Imaging of Meckel's diverticulum in adults: pictorial essay. Clin Imaging 2014;38:557–64. 24998882 10.1016/j.clinimag.2014.04.020

[7] Won Y, Lee HW, Ku YM, Lee SL, Seo KJ, Lee JI, et al. Multidetector-row computed tomography (MDCT) features of small bowel obstruction (SBO) caused by Meckel's diverticulum. Diagn Interv Imaging 2016;97:227–32. 26493762 10.1016/j.diii.2015.09.006

[8] Lekias JS. Bowel obstruction due to Meckel's diverticulum. Med J Aust 1958;45:37–40. 13503415 10.5694/j.1326-5377.1958.tb85988.x

